# Dietary intake and hospitalisation due to diabetic ketoacidosis and hypoglycaemia in individuals with type 1 diabetes

**DOI:** 10.1038/s41598-021-81180-0

**Published:** 2021-01-15

**Authors:** Aila J. Ahola, Valma Harjutsalo, Merlin C. Thomas, Carol Forsblom, Per-Henrik Groop

**Affiliations:** 1 Folkhälsan Institute of Genetics, Folkhälsan Research Center, Helsinki, Finland; 2 Abdominal Center, Nephrology, Helsinki University Hospital, University of Helsinki, Helsinki, Finland; 3 Research Program for Clinical and Molecular Metabolism, Faculty of Medicine, University of Helsinki, Helsinki, Finland; 4 National Institute for Health and Welfare, Helsinki, Finland; 5 Department of Diabetes, Central Clinical School, Monash University, Melbourne, VIC Australia

**Keywords:** Diseases, Risk factors

## Abstract

We investigated the association between diet and risk of hospitalisation for diabetic ketoacidosis (DKA) or hypoglycaemia in type 1 diabetes. Food records were used to assess dietary intake. Data on DKA and hypoglycaemia hospitalisations, within two years of dietary assessments, were obtained from registries. Analyses were conducted with and without macronutrient substitution. Data were available from 1391 participants, 28 (2.0%) and 55 (4.0%) of whom were hospitalised due to DKA or hypoglycaemia, respectively. In the adjusted model, self-reported alcohol intake was associated with increased (per 10 g: B = 1.463, 95% CI = 1.114–1.922, *p* = 0.006; per E%: B = 1.113, 95% CI = 1.027–1.206, *p* = 0.009), and fibre intake with reduced (per g/MJ: B = 0.934, 95% CI = 0.878–0.995, *p* = 0.034) risk of DKA hospitalisation. Substituting carbohydrates for fats was associated with increased risk for hypoglycaemia hospitalisation (B = 1.361, 95% CI = 1.031–1.795, *p* = 0.029), while substituting alcohol for carbohydrates (B = 1.644, 95% CI = 1.006–2.685, *p* = 0.047) or proteins (B = 2.278, 95% CI = 1.038–4.999, *p* = 0.040) increased the risk for DKA hospitalisation. In conclusion, refraining from alcohol intake is a preventable risk factor for DKA, while higher fibre intake seems rather protective. Increasing carbohydrate intake while decreasing that of fats, is associated with higher hypoglycaemia risk. Whether this is a cause or effect of hypoglycaemia remains to be established.

## Introduction

Diabetic ketoacidosis (DKA) and severe hypoglycaemia are important medical events and potentially life-threatening acute complications of type 1 diabetes. Associated with symptoms such as disorientation, convulsions, or even loss of consciousness, severe hypoglycaemia prevents the affected individual from taking corrective actions to normalize glycaemia. Importantly, while only a fraction of patients account for the most episodes of severe hypoglycaemia^1^, their emergence may provoke cardiovascular events^2^. DKA, instead, characterized by hyperglycaemia, acidosis, and the presence of ketone bodies, commonly requires hospitalization in order to, not only correct the hyperglycaemia, but also the associated dehydration and electrolyte imbalances.

With optimal diabetes management, both DKA and severe hypoglycaemia are potentially preventable complications. Although adherence with insulin injections^3–5^ is the most important factor, diet and lifestyle may also significantly contribute to the risks for DKA and hypoglycaemia. For example, excessive alcohol intake can be a risk factor for DKA and severe hypoglycaemia^6–8^. Besides the studies on alcohol, we are aware of only a handful of studies of other dietary constituents^9–11^. In an early paper, Clarke et al.^9^ investigated the association between a number of self-management practices and the history of severe hypoglycaemia in 93 adult participants with type 1 diabetes. In their study, prior to every blood glucose measurement, participants were instructed to report whether their most recent exercise activity, insulin dose, and food intake was more than, less than, or the same as usual. With this crude level of assessing health behaviour, authors could not distinguish between individuals with and without history of severe hypoglycaemia. However, food intake at the level of “lower than usual” significantly predicted blood glucose readings of < 3.9 mmol/l. In a small sample of adolescents with type 1 diabetes, Johns et al.^10^ reported that individuals with and without any diabetes-related hospitalizations, over the past year, had comparable macronutrient intakes. Those with such hospitalizations, however, reported lower energy intake compared to those with no hospitalizations. In their recent contribution, Zhong et al.^11^ investigated the association between dietary intake and the risk of non-severe hypoglycaemia in 98 adolescents with type 1 diabetes. In that study, diet was assessed with two 24-h recalls, and hypoglycaemia was defined as blood glucose concentration < 70 mg/dl for at least 10 min without the need for external assistance to manage glycaemia. In their study, higher intakes of soluble fibre and protein were associated with both daytime and nocturnal hypoglycaemia, while glycaemic index and the intakes of monounsaturated and polyunsaturated fatty acids were protective of daytime hypoglycaemia. Importantly, the above studies have greatly varied with respect to the methods used to assess dietary intake and acute complications.

In this cross-sectional study, we investigated the associations between dietary macronutrient and fibre intakes and hospitalisations due to DKA and severe hypoglycaemia in a large sample of adult individuals with type 1 diabetes.

## Results

Data were available from 1391 adults with type 1 diabetes (44% men, median age 49 years). Altogether, 28 (2.0%) participants requiring hospital treatment for DKA were identified (Table 1). There were 35 episodes of DKA requiring hospital treatment. In all, 55 (4.0%) participants required hospital treatment for hypoglycaemia over the same time period, and overall there were 68 hospitalisations for hypoglycaemia. Of the total sample, we identified only one individual with both DKA and hypoglycaemia hospitalisation.

**Table 1:** Participant characteristics divided by the hospitalisation status.

	No DKA hospitalisations	DKA hospitalisations	No hypo hospitalisations	Hypo hospitalisations
	n = 1363 (98%)	n = 28 (2.0%)	n = 1336 (96%)	n = 55 (4.0%)
**Hospitalisations**				
n = 1	Na	23	Na	47
n = 2	Na	4	Na	5
n = 3	Na	0	Na	2
n = 4	Na	1	Na	0
n = 5	Na	0	Na	1
Men, n (%)	601 (44)	8 (29)	581 (44)	28 (51)
Age, years	49 (39–58)	52 (42–58)	48 (39–58)	53 (43–61)
Age at diagnosis, years	16 (10–26)	21 (12–26)	16 (10–26)	16 (11–27)
Current smoker, n (%)	162 (12)	7 (26)	157 (12)	12 (22)*
Physical activity, METh	5.0 (2.8–8.2)	4.7 (2.3–6.9)	5.0 (2.8–8.2)	3.9 (1.8–9.4)
Insulin dosing, IU/kg	0.6 (0.5–0.7)	0.6 (0.5–0.6)	0.6 (0.5–0.7)	0.6 (0.4–0.7)
BDI score ≥ 16, n (%)	104 (11)	2 (9.5)	103 (11)	3 (8.1)
Low SES, n (%)	133 (11)	3 (12)	128 (11)	8 (17)
Coeliac disease, n (%)	50 (3.7)	1 (3.6)	49 (3.7)	2 (3.6)
Thyroid medication, n (%)	210 (16)	3 (11)	205 (16)	8 (15)
HbA_1c_, mmol/mol	64 (56–72)	71 (66–77)**	64 (56–73)	66 (59–73)
HbA_1c_, %	8.0 (7.3–8.7)	8.6 (8.2–9.2)**	8.0 (7.3–8.8)	8.2 (7.5–8.8)
SBP, mmHg	135 (124–149)	136 (126–152)	135 (123–149)	142 (126–150)
DBP, mmHg	77 (70–84)	84 (74–89)*	77 (70–84)	78 (70–85)
Triglycerides, mmol/l	0.94 (0.71–1.30)	1.10 (0.79–1.65)	0.94 (0.71–1.31)	0.92 (0.77–1.25)
Total cholesterol, mmol/l	4.5 (4.0–5.1)	6.7 (4.0–5.1)	4.5 (4.0–5.1)	4.2 (3.6–4.7)**
HDL-cholesterol, mmol/l	1.6 (1.3–1.9)	1.5 (1.3–1.8)	1.6 (1.3–1.9)	1.5 (1.2–1.8)
eGFR, ml/min/1.73m^2^	97 (79–109)	99 (58–108)	97 (79–109)	92 (54–107)*

Data are presented as frequency for categorical variables, and median (interquartile range) for continuous variables with skewed distribution. Using Chi-squared test and Mann–Whitney U-test, for the respective variable types, we compared those with each hospitalisation type and those with no hospitalisations.

*DKA* diabetic ketoacidosis, *Hypo* hypoglycaemia, *METh* metabolic equivalent of task hour, *BDI* Beck Depression Inventory, scores ≥ 16 are indicative of signs of depression, *Low SES* low socioeconomic status, unskilled blue collar workers, *SBP* systolic blood pressure, *DBP* diastolic blood pressure, *eGFR* estimated glomerular filtration rate.

*p < 0.05; **p < 0.01.

### Predictors of hospitalisation for diabetic ketoacidosis

Compared to the individuals without, those with DKA hospitalisations had worse glycaemic control and higher diastolic blood pressure (Table 1). However, participants differing in the DKA hospitalisation status were comparable with respect to the energy, macronutrient, and fibre intakes (Table 2). In the adjusted model, self-reported fibre intake was negatively associated with the risk of DKA hospitalisation (in g/MJ: B = 0.934, 95% Confidence Interval, CI = 0.878–0.995, *p* = 0.034) (Table 3). Alcohol intake, instead, was positively associated with DKA hospitalisation (per 10 g: B = 1.463, CI = 1.114–1.922, *p* = 0.006; in percentages of total energy intake, E%: B = 1.113, 95% CI = 1.027–1.206, *p* = 0.009). Although individuals in the highest decile of alcohol intake had more than threefold increased risk of DKA hospitalisations (in grams: B = 3.527, 95% CI = 1.219–10.205, *p* = 0.020; in E%: B = 3.165, 95% CI = 1.098–9.120, *p* = 0.033), the relationship was linear such that even modest alcohol intake was associated with an increased risk. In the macronutrient substitution analyses, increasing alcohol intake at the expense of either carbohydrates (B = 1.644, 95% CI = 1.006–2.685, *p* = 0.047) or proteins (B = 2.278, 95% CI = 1.038–4.999, *p* = 0.040) was associated with higher DKA hospitalisation risk (Table 4).

**Table 2:** Energy, macronutrient, and fibre intakes of the participants divided by hospitalisations.

	No DKA hospitalisations	DKA hospitalisations	No hypo hospitalisations	Hypo hospitalisations
	n = 1363 (98%)	n = 28 (2.0%)	n = 1336 (96%)	n = 55 (4.0%)
Energy, kJ	7760 (6582–9161)	7213 (6259–8854)	7753 (6582–9139)	7441 (6251–9341)
Carbohydrates, g	201 (166–241)	181 (145–221)	201 (165–241)	200 (170–234)
Carbohydrates, E%	43.7 (39.2–48.3)	41.5 (38.4–45.2)	43.6 (39.0–48.1)	45.5 (41.3–49.0)*
Fibre, g/MJ	2.8 (2.2–3.5)	2.8 (2.1–3.2)	2.8 (2.2–3.5)	3.0 (2.4–3.3)
Fats, g	73 (59–90)	70 (56–87)	73 (59–90)	66 (54–78)*
Fats, E%	35.4 (31.2–39.4)	36.6 (32.3–41.9)	35.4 (31.3–39.5)	34.2 (30.1–37.4)
Proteins, g	77 (65–92)	70 (62–85)	77 (65–92)	74 (63–91)
Proteins, E%	16.5 (14.8–18.5)	16.1 (14.3–18.3)	16.5 (14.8–18.4)	16.5 (14.6–19.2)
Protein, g/kg	1.1 (0.9–1.3)	1.0 (0.8–1.3)	1.1 (0.9–1.3)	1.1 (0.8–1.3)
Alcohol, g	1.9 (0–7.9)	4.1 (0–14.8)	2.0 (0–8.2)	1.9 (0–7.3)
Alcohol, E%	0.8 (0–3.0)	1.7 (0–5.2)	0.8 (0–3.0)	1.0 (0–3.1)

Data are presented as median (interquartile range) for continuous variables with skewed distribution. Mann–Whitney U-test was used for comparing those with each hospitalisation type and those with no hospitalisations.

*DKA* diabetic ketoacidosis, *Hypo* hypoglycaemia, *E%* percentage of total energy intake.

*p < 0.05.

**Table 3:** Associations between macronutrient and fibre intakes and acute hospitalisations due to ketoacidosis and hypoglycaemia.

	DKA			Hypoglycaemia		
	B	95% CI	*p*	B	95% CI	*p*
Carbohydrates, g	0.993	0.982–1.004	0.208	1.007	0.998–1.016	0.132
Carbohydrates, E%	0.970	0.920–1.022	0.280	1.036	0.994–1.079	0.097
Fibre, g/MJ	0.934	0.878–0.995	0.034	1.011	0.970–1.053	0.599
Fats, g	1.013	0.985–1.041	0.367	0.984	0.963–1.006	0.146
Fats, E%	1.025	0.967–1.085	0.406	0.965	0.922–1.009	0.118
Proteins, g	0.974	0.944–1.005	0.105	1.004	0.983–1.025	0.701
Proteins, E%	0.907	0.787–1.045	0.176	1.022	0.928–1.126	0.657
Protein, g/kg	0.629	0.136–2.907	0.553	1.688	0.556–5.126	0.356
Alcohol, 10 g	1.463	1.114–1.922	0.006	0.994	0.743–1.330	0.966
Alcohol, E%	1.113	1.027–1.206	0.009	0.994	0.914–1.080	0.882

Logistic regression analysis. Models are adjusted for age, sex, insulin dosing per weight in kg, physical activity, and total energy intake.

*DKA* diabetic ketoacidosis, *E%* percentage of total energy intake.

**Table 4:** Associations between macronutrient substitutions and acute hospitalisations due to ketoacidosis and hypoglycaemia.

Macronutrients	DKA			Hypoglycaemia		
Index (excluded)	B	95% CI	*p*	B	95% CI	*p*
CHO (fat)	0.719	0.494–1.048	0.087	1.361	1.031–1.795	0.029
Protein (CHO)	0.722	0.315–1.653	0.441	0.969	0.553–1.699	0.913
Protein (fat)	0.517	0.212–1.257	0.145	1.312	0.723–2.380	0.371
Alcohol (CHO)	1.644	1.006–2.685	0.047	0.965	0.599–1.555	0.885
Alcohol (protein)	2.278	1.038–4.999	0.040	1.122	0.596–2.113	0.722
Alcohol (fat)	1.192	0.680–2.089	0.540	1.296	0.764–2.197	0.337

Logistic regression analysis. For DKA, the models are adjusted for age, sex, insulin dosing per weight in kg, physical activity, HbA_1c_, and diastolic blood pressure; for hypoglycaemia, the adjustments were made for age, sex, insulin dosing, physical activity, smoking, total cholesterol concentration, and estimated glomerular filtration rate. In these substitution models, one of the macronutrients (shown in the parentheses) is left out, while one of the macronutrients is the index macronutrient. The beta value represents an increase or decrease in the risk of diabetic ketoacidosis or hypoglycaemia hospitalisation when the intake of the index macronutrient is increased by 5% at the expense of the excluded macronutrient.

*DKA* diabetic ketoacidosis, *CHO* carbohydrate.

### Predictors of hospitalisation for hypoglycaemia

Participants that required hospitalisation for hypoglycaemia were more frequently smokers, and had lower total cholesterol concentration and eGFR than individuals who did not experience a severe hypoglycaemic event requiring hospitalisation (Table 1). Additionally, participants with hypoglycaemia hospitalisations had higher median carbohydrate (E%), and lower fat (g) intake (Table 2). No single macronutrient or fibre intake was associated with the risk of hypoglycaemia hospitalisation (Table 3). However, in the macronutrient substitution model, substituting carbohydrates for fats was associated with a modestly increased risk of severe hypoglycaemia requiring hospitalisation (B = 1.361, 95% CI = 1.031–1.795, *p* = 0.029) (Table 4).

### Repeatability of the diet recordings

A total of 69 participants had a two diet recordings within a 4 year time period. Besides of being older (51 years vs. 47 years, *p* = 0.030), individuals with two recordings were otherwise comparable with those with one recording (Supplementary Table S1). The median (interquartile range) of the time gap between the two diet recordings was 3.6 (2.7–3.8) years. None of the mean paired differences of the dietary variables, between the two diet recordings, were statistically significant (Supplementary Table S2).

## Discussion

Severe hypoglycaemia and DKA are important acute complications of type 1 diabetes. After ischemic heart disease, hypoglycaemic coma and DKA are the next most common causes of death in individuals with type 1 diabetes younger than 50 years of age^12^. Although diet and lifestyle intervention to prevent hyperglycaemia are prioritised in individuals with type 1 diabetes, the role of diet and lifestyle modification to prevent hypoglycaemia or DKA remain poorly understood. In this study, we show that alcohol and fibre intake, as well as dietary macronutrient composition may play important roles in the development of DKA and severe hypoglycaemia, respectively.

In Finnish adults with type 1 diabetes, increasing the intake of carbohydrates and reducing that of fats were associated with increased risk of severe hypoglycaemia requiring hospitalisations. Importantly, these associations remained independent after adjusting for a number of factors including insulin dosing and physical activity. While this observation may not go in line with the dietary recommendations, it has to be acknowledged that the results could also be a reflection of individuals prone to experience hypoglycaemia trying to prevent new episodes with higher carbohydrate intake in relation to that of fat. The use of high carbohydrate, low fat diet to prevent hypoglycaemias could, however, be counterintuitive as a change from a standard diet to a low-carbohydrate diet rather has the potential to reduce the incidence of hypoglycaemias^13,14^. Importantly, while increased carbohydrate intake and the associated higher insulin doses give rise to larger variations in blood glucose concentrations^13,15^, marked reductions in glycaemic variability have been reported in studies of low-carbohydrate diets^14,16^. High variability of the blood glucose levels, on the other hand, is a well-known risk factor for hypoglycaemia^5,17^, a phenomenon that could also explain the current observations. Fat intake, on the other hand, does not raise the blood glucose levels and, following the above reasoning and the current observations, would reduce the hypoglycaemia risk. Importantly, previous small studies have not identified differences in dietary intake in individuals with and without risk of severe hypoglycaemia^9,10^. Zhong et al. suggested, however, that higher intakes of soluble fibre and protein were associated with daytime and nocturnal hypoglycaemia, while glycaemic index and the intakes of monounsaturated and polyunsaturated fatty acids were protective of daytime hypoglycaemia in adolescents with type 1 diabetes^11^. Overall, there is a shortage of large contemporary studies of the association between dietary intake and hypoglycaemia requiring hospitalisation in adults with type 1 diabetes such as the one presented in this paper.

Excessive alcohol intake is known to be associated with ketoacidosis. In a population of almost 30,000 young individuals with type 1 diabetes, for example, Hermann et al. observed that the adjusted DKA rate was significantly increased in those with high alcohol intakes^6^. In another study, a diagnosis of substance or alcohol abuse was noted in 44% of DKA-related deaths in middle aged subjects^18^. In our study, even a small self-reported intake of alcohol measured either as absolute intake or as percentage of total energy intake, was associated with an increased risk of DKA. This may reflect the relationship between self-reported alcohol intake and the frequency of binge drinking, although even small amounts of alcohol may affect hepatic metabolism^19^. Importantly, alcohol consumption may lead to reduced adherence to diabetes self-management practices and thereby increase the risk of acute complications. Although recommendations of abstinence are often levelled at individuals at risk of hypoglycaemia, and alcohol consumption has been previously associated with hypoglycaemia^7^, in our study the major benefit of alcohol abstinence may be in those with poor glucose control at risk for DKA. Beyond alcohol consumption, abundant intake of fibres intake was associated with reduced risk for DKA hospitalisation, in the current study. While we are not aware of prior studies showing such a protective effect, there is some indication that high fibre intake may be beneficial for blood glucose control^20,21^ and, thus, could also impact the DKA risk.

A number of limitations are worthy of mention. As the study was observational and cross sectional data were collected only at one time point, it limits us from making causal inferences. While the role of alcohol consumption in the risk of DKA is fairly well established, our observations linking high carbohydrate and low fat intake to severe hypoglycaemia may also be explained by a reverse causation. It should also be noted that the dietary intake was self-reported. Importantly, data collected with self-report methods may be subject to misreporting. Whether individuals prone to develop severe hypoglycaemia or DKA would systematically differ in misreporting from those in low risk is however not known. Importantly, individuals with and without hospitalisations were comparable with respect to many background variables, including socioeconomic status, depressive symptomatology, and physical activity. Dietary intake was also only reported at one point, in the middle of the 4-year observation period for the hospitalizations. In a small sample of individuals with diet recording repeated within a 4-year time frame, however, we observed no difference in energy, fibre, or macronutrient intake, suggesting that during the selected (two-sided) 2-year time period no major changes in dietary intake took place. It should also be noted that data on a number of important confounders, including hypoglycaemia awareness, family support, and type of insulin used, were not available for the analyses. In the current study, DKA and severe hypoglycaemia were identified from hospitalisation registries. With the use of such registries we were able to reliably identify all severe cases that needed acute inpatient care. However with this method we likely missed a number of milder cases that were treated in outpatient units or by friends and family members, and therefore reduced power to identify associations between dietary exposures and acute complications. Finally, as the study participants were volunteers, individuals less interested in diet and health-related matters may have been under-represented in this sample. The potential under-representation of individuals with less healthy life-styles and dietary practices would likely have diluted the phenomenon taking place in the real-world. With these limitations in mind, the current study with a large number of well-defined individuals with type 1 diabetes is an important addition to the limited pool of knowledge on the association between dietary intake and acute complications of type 1 diabetes.

In conclusion, supported by the current dietary guidelines, alcohol intake was associated with an increased DKA admission risk, and abundant intake of fibres rather reduced the risk. Increasing carbohydrate intake at the expense of fat, instead, was associated with increased risk of hypoglycaemia hospitalisations. More studies are needed to reveal the associations between diet and acute complications of type 1 diabetes.

## Methods

Participants were individuals with type 1 diabetes taking part in the ongoing nation-wide Finnish Diabetic Nephropathy (FinnDiane) Study. In the FinnDiane Study, included are individuals with the onset of diabetes prior to the age of 40 years and permanent insulin treatment initiated within a year from the diabetes diagnosis. In the current analyses, all adult FinnDiane Study participants, investigated between August 2007 and January 2019, with a completed food record as described in more detail below, were included. The Ethics Committee of Helsinki and Uusimaa Hospital District approved the study protocol (Ethics committee reference number 491/E5/06). The study was carried out in accordance with the Declaration of Helsinki and its later amendments. All participants signed written informed consent prior to study participation.

Cross-sectional data from 1391 individuals participating in the FinnDiane study, in whom records of dietary intake, data on smoking habits, alcohol intake, insulin dosing, physical activity, and social class, using a self-reported patient questionnaire, were included. The reported insulin dose was divided by weight in kg. Based on the reported duration and intensity of physical activity, metabolic equivalent of task hours (METh) was calculated for each participant. Unskilled blue collar workers were classified as having low socioeconomic status. Depression was assessed using Beck Depression Inventory (BDI)^22^, as previously described^23^. Individuals with BDI scores ≥ 16 were considered having symptoms of depression. Details on clinical status, including age at diagnosis, insulin therapy and other regular medications, together with presence, severity and management of diabetic complications including diabetic retinopathy and macrovascular disease, were obtained from medical records by the attending physician using a standardised questionnaire. Blood pressure measurements were performed twice in the sitting position, with a ten-minute interval between testing, with the mean of the two measurements was calculated. Participants’ height and weight were measured while wearing light clothing. Fasting blood samples were obtained for the measurement of HbA_1c_, lipids and creatinine, assayed at the laboratory of the Helsinki University Hospital. HbA_1c_ was assessed locally using a standardized assay. The glomerular filtration rate (GFR) was estimated using the CKD-EPI formula^24^.

The methods to study dietary intake, in the FinnDiane Study, have been previously described in more detail^25^. The study visit took place at around the same time as the diet assessment. In short, participants first filled out a validated diet questionnaire and thereafter completed two 3-day diet records with 2 to 3 months’ interval. The dates for these 3 days were allocated and covered consecutive days either from Thursday to Saturday or Sunday to Tuesday. In order to capture variation in the dietary intake, the other set of days (Sunday to Tuesday or Thursday to Saturday, respectively) was allocated for the second recording. For the purpose of the current study, data collected with the diet records were only used. AivoDiet software (version 2.0.2.3, AIVO, Turku, Finland) was used to calculate the mean energy, macronutrient, and fibre intakes from the 3 or 6 (if available) days’ recordings. For the analyses, we only included participants with plausible energy intake (5.0–14.6 MJ). To test the repeatability of the diet recording over the years, we additionally identified individuals with two diet recordings within a 4 year time window.

Data on DKA and hypoglycaemia hospitalisations, for the above mentioned FinnDiane Study participants, were obtained from the Finnish nation-wide health registries. Only events that occurred in a four-year window, two years prior and two years after the formal assessment of the dietary intake are reported in this study.

### Statistical analyses

Data on categorical variables are presented as frequencies, while continuous variables with skewed distribution are shown as medians (interquartile ranges). Between-group comparisons were performed with Chi-squared test and Mann–Whitney U-test, respectively. The associations between macronutrient intakes and hospitalisations were studied with a Firth’s logistic regression analysis, the method for rare events^26^, where macronutrient data were entered either as grams or percentages of energy intake. In addition, we applied macronutrient substitution where one macronutrient at a time was excluded from the model. Subsequent significant observation would refer to increase or decrease in the risk of hospitalisation related to a 5 E% increase in the index macronutrient at the expense of the macronutrient that was excluded from the model. In the models, we included age, sex, insulin dosing, physical activity, and total energy intake for both endpoints. For the purpose of studying the repeatability of the diet records, we used paired samples t-test. Analyses were done using IBM SPSS Statistics for Windows, Version 25.0 (IBM, Armonk, NY, USA). A two-tailed *p* value < 0.05 indicated statistical significance.

## Supplementary Information

Supplementary Information.
