# Supplementary material for: Dietary intake and hospitalisation due to diabetic ketoacidosis and hypoglycaemia in individuals with type 1 diabetes
Source: Sci Rep. 2021 Jan 15;11:1638. doi: 10.1038/s41598-021-81180-0 (PMC7810746; doi:10.1038/s41598-021-81180-0)
Supplement: Supplementary file 1 — Supplementary Information. [file 41598_2021_81180_MOESM1_ESM.pdf]

**Scientific Reports**

# Dietary intake and hospitalisation due to diabetic ketoacidosis and hypoglycaemia in individuals with type 1 diabetes

Aila J. Ahola[1,2,3], Valma Harjutsalo[1,2,3,4], Merlin C. Thomas[5], Carol Forsblom[1,2,3], Per-Henrik Groop[1,2,3,5*]

[1]Folkhälsan Institute of Genetics, Folkhälsan Research Center, Helsinki, Finland

[2]Abdominal Center, Nephrology, University of Helsinki and Helsinki University Central Hospital, Helsinki, Finland

[3]Research Program for Clinical and Molecular Metabolism, Faculty of Medicine, University of Helsinki, Finland

[4]National Institute for Health and Welfare, Helsinki, Finland

[5]Department of Diabetes, Central Clinical School, Monash University, Melbourne, Victoria, Australia.

*Corresponding author: Per-Henrik Groop, Folkhälsan Research Center, Biomedicum Helsinki C318b, PO Box 63, FI-00014 University of Helsinki, Finland. Tel +358 500 430 436. Email: per-henrik.groop@helsinki.fi

**Supplementary Table S1** Participant characteristics of individuals with and without two diet recordings within a 4 year time period

| | With two diet recordings | With one diet recording | P |
|---|---|---|---|
| | n = 69 (5.0%) | n = 1322 (95.0%) | |
| Men, n (%) | 35 (50.7) | 574 (43.4) | 0.263 |
| Age, years | 51 (40 – 61) | 47 (37 – 56) | 0.030 |
| Age at diagnosis, years | 18 (10 – 25) | 16 (10 – 26) | 0.617 |
| Current smoker, n (%) | 7 (10.6) | 162 (12.7) | 0.849 |
| $HbA_{1c}$, mmol/mol | 65 (56 – 77) | 64 (55 – 72) | 0.176 |
| $HbA_{1c}$, % | 81 (7.3 – 9.2) | 8.0 (7.2 – 8.7) | 0.176 |
| SBP, mmHg | 139 ± 19 | 137 ± 18 | 0.357 |
| DBP, mmHg | 77 (71 – 84) | 77 (70 – 84) | 0.831 |
| Triglycerides, mmol/l | 0.97 (0.77 – 1.46) | 0.94 (0.71 – 1.30) | 0.232 |
| Total cholesterol, mmol/l | 4.5 (4.0 – 5.2) | 4.5 (4.0 – 5.1) | 0.990 |
| HDL-cholesterol, mmol/l | 1.5 (1.3 – 1.8) | 1.6 (1.3 – 1.9) | 0.135 |
| eGFR, ml/min/1.73$m^2$ | 99 (79 – 112) | 97 (78 – 109) | 0.299 |

Data are presented as frequency for categorical variables, median (interquartile range) for continuous variables with skewed distribution, and mean ± standard deviation for continuous variables with normal distribution. Using Chi-squared test, Mann-Whitney U-test, and independent samples' t-test, for the respective variable types, we compared those with one and two diet recordings. SBP, systolic blood pressure; DBP, diastolic blood pressure; eGFR, estimated glomerular filtration rate.

**Supplementary Table S2** Repeatability of dietary intake in individuals with two diet recordings within a 4 year time period

| | Mean paired differences | 95% Confidence Interval of the difference | P |
|---|---|---|---|
| Energy, kJ | 48.2 | -297.5 – 394.0 | 0.782 |
| Carbohydrates, g | 5.4 | -6.2 – 17.0 | 0.357 |
| Carbohydrates, E% | 0.7 | -0.7 – 2.1 | 0.301 |
| Fibre, g/MJ | 1.4 | -0.1 – 3.0 | 0.065 |
| Fats, g | -1.2 | -6.0 – 3.7 | 0.635 |
| Fats, E% | -0.7 | -2.1 – 0.7 | 0.343 |
| Proteins, g | -1.6 | -5.1 – 1.9 | 0.368 |
| Proteins, E% | -0.4 | -1.0 – 0.2 | 0.155 |
| Alcohol, g | 1.3 | -0.4 – 3.1 | 0.120 |
| Alcohol, E% | 0.5 | -0.1 – 1.1 | 0.094 |

Paired sample t-test. E%, percentage of total energy intake.

## Supplementary information of The Finnish Diabetic Nephropathy Study Centers

| | |
|---|---|
| Anjalankoski Health Center | S.Koivula, T.Uggeldahl |
| Central Finland Central Hospital, Jyväskylä | T.Forslund, A.Halonen, A.Koistinen, P.Koskiaho, M.Laukkanen, J.Saltevo, M.Tiihonen |
| Central Hospital of Åland Islands, Mariehamn | M.Forsen, H.Granlund, A.-C.Jonsson, B.Nyroos |
| Central Hospital of Kanta-Häme, Hämeenlinna | P.Kinnunen, A.Orvola, T.Salonen, A.Vähänen |
| Central Hospital of Kymenlaakso, Kotka | R.Paldanius, M.Riihelä, L.Ryysy |
| Central Hospital of Länsi-Pohja, Kemi | H.Laukkanen, P.Nyländen, A.Sademies |
| Central Ostrobothnian Hospital District, Kokkola | S.Anderson, B.Asplund, U.Byskata, P.Liedes, M.Kuusela, T.Virkkala |
| City of Espoo Health Center: | |
| Espoonlahti | A.Nikkola, E.Ritola |
| Tapiola | M.Niska, H.Saarinen |
| Samaria | E.Oukko-Ruponen, T.Virtanen |
| Viherlaakso | A.Lyytinen |
| City of Helsinki Health Center: | |
| Puistola | H.Kari, T.Simonen |
| Suutarila | A.Kaprio, J.Kärkkäinen, B.Rantaeskola |
| Töölö | P.Kääriäinen, J.Haaga, A-L.Pietiläinen |
| City of Hyvinkää Health Center | S.Klemetti, T.Nyandoto, E.Rontu, S.Satuli-Autere |
| City of Vantaa Health Center: | |
| Korso | R.Toivonen, H.Virtanen |
| Länsimäki | R.Ahonen, M.Ivaska-Suomela, A.Jauhiainen |
| Martinlaakso | M.Laine, T.Pellonpää, R.Puranen |
| Myyrmäki | A.Airas, J.Laakso, K.Rautavaara |
| Rekola | M.Erola, E.Jatkola |
| Tikkurila | R.Lönnblad, A.Malm, J.Mäkelä, E.Rautamo |
| Heinola Health Center | P.Hentunen, J.Lagerstam |
| Helsinki University Central Hospital, Department of Medicine, Division of Nephrology | M.Feodoroff, D.Gordin, O.Heikkilä, K.Hietala, J.Fagerudd, M.Korolainen, L.Kyllönen, J.Kytö, S.Lindh, K.Pettersson-Fernholm, M.Rosengård-Bärlund, A.Sandelin, L.Thorn, J.Tuomikangas, T.Vesisenaho, J.Wadén |
| Herttoniemi Hospital, Helsinki | V.Sipilä |
| Hospital of Lounais-Häme, Forssa | T.Kalliomäki, J.Koskelainen, R.Nikkanen, N.Savolainen, H.Sulonen, E.Valtonen |
| Hyvinkää Hospital | L. Norvio, A.Hämäläinen |
| Iisalmi Hospital | E.Toivanen |
| Jokilaakso Hospital, Jämsä | A.Parta, I.Pirttiniemi |
| Jorvi Hospital, Helsinki University Central Hospital | S.Aranko, S.Ervasti, R.Kauppinen-Mäkelin, A.Kuusisto, T.Leppälä, K.Nikkilä, L.Pekkonen |
| Jyväskylä Health Center, Kyllö | K.Nuorva, M.Tiihonen |
| Kainuu Central Hospital, Kajaani | S.Jokelainen, K.Kananen, M.Karjalainen, P.Kemppainen, A-M.Mankinen, |
| A.Reponen | M.Sankari |
| Kerava Health Center | H.Stuckey, P.Suominen |
| Kirkkonummi Health Center | A.Lappalainen, M.Liimatainen, J.Santaholma |
| Kivelä Hospital, Helsinki | A.Aimolahti, E.Huovinen |
| Koskela Hospital, Helsinki | V.Ilkka, M.Lehtimäki |
| Kotka Health Center | E.Pälikkö-Kontinen, A.Vanhanen |
| Kouvola Health Center | E.Koskinen, T.Siitonen |
| Kuopio University Hospital | E.Huttunen, R.Ikäheimo, P.Karhapää, P.Kekäläinen, M.Laakso, T.Lakka, E.Lampainen, L.Moilanen, S. Tanskanen L.Niskanen, U.Tuovinen, I.Vauhkonen, E.Voutilainen |
| Kuusamo Health Center | T.Kääriäinen, E.Isopoussu |
| Kuusankoski Hospital | E.Kilkki, I.Koskinen, L.Riihelä |
| Laakso Hospital, Helsinki | T.Meriläinen, P.Poukka, R.Savolainen, N.Uhlenius |
| Lahti City Hospital | A.Mäkelä, M.Tanner |
| Lapland Central Hospital, Rovaniemi | L.Hyvärinen, K.Lampela, S.Pöykkö, T.Rompasaari, S.Severinkangas, T.Tulokas |
| Lappeenranta Health Center | P. Erola, L.Härkönen, P.Linkola, T.Pekkanen, I.Pulli, E.Repo |
| Lohja Hospital | T.Granlund, K.Hietanen, M.Porrassalmi, M.Saari, T.Salonen, M.Tiikkainen, |
| Länsi-Uusimaa Hospital, Tammisaari | I.-M.Jousmaa, J.Rinne |
| Loimaa Health Center | A.Mäkelä, P.Eloranta |
| Malmi Hospital, Helsinki | H.Lanki, S.Moilanen, M.Tilly-Kiesi |
| Mikkeli Central Hospital | A.Gynther, R.Manninen, P.Nironen, M.Salminen, T.Vänttinen |
| Mänttä Regional Hospital | I.Pirttiniemi, A-M.Hänninen |
| North Karelian Hospital, Joensuu | U-M.Henttula, P.Kekäläinen, M.Pietarinen, A.Rissanen, M.Voutilainen |
| Nurmijärvi Health Center | A.Burgos, K.Urtamo |
| Oulaskangas Hospital, Oulainen | E.Jokelainen, P-L.Jylkkä, E.Kaarlela, J.Vuolaspuro |
| Oulu Health Center | L.Hiltunen, R.Häkkinen, S.Keinänen-Kiukaanniemi |

| | |
|---|---|
| Oulu University Hospital | R.Ikäheimo |
| Päijät-Häme Central Hospital | H.Haapamäki, A.Helanterä, S.Hämäläinen, V.Ilvesmäki, H.Miettinen |
| Palokka Health Center | P.Sopanen, L.Welling |
| Pieksämäki Hospital | V.Sevtsenko, M.Tamminen |
| Pietarsaari Hospital | M-L.Holmbäck, B.Isomaa, L.Sarelin |
| Pori City Hospital | P.Ahonen, P.Merisalo, E.Muurinen, K.Sävelä |
| Porvoo Hospital | M.Kallio, B.Rask, S.Rämö |
| Raahe Hospital | A.Holma, M.Honkala, A.Tuomivaara, R.Vainionpää |
| Rauma Hospital | K.Laine, K.Saarinen, T.Salminen |
| Riihimäki Hospital | P.Aalto, E.Immonen, L.Juurinen |
| Salo Hospital | A.Alanko, J.Lapinleimu, P.Rautio, M.Virtanen |
| Satakunta Central Hospital, Pori | M.Asola, M.Juhola, P.Kunelius, M.-L.Lahdenmäki, P.Pääkkönen, M.Rautavirta |
| Savonlinna Central Hospital | T.Pulli, P.Sallinen, M.Taskinen, E.Tolvanen, T.Tuominen H.Valtonen, A.Vartia, S-L.Viitanen |
| Seinäjoki Central Hospital | O.Antila, E.Korpi-Hyövälti, T.Latvala, E.Leijala, T.Leikkari, M.Punkari N.Rantamäki, H.Vähävuori |
| South Karelia Central Hospital, Lappeenranta | T.Ensala, E.Hussi, R.Härkönen, U.Nyholm, J.Toivanen |
| Tampere Health Center | A.Vaden, P.Alarotu, E.Kujansuu, H.Kirkkopelto-Jokinen, M.Helin, S.Gummerus, L.Calonius, T.Niskanen, T.Kaitala, T.Vatanen |
| Tampere University Hospital | P. Hannula, I.Ala-Houhala, R.Kannisto, T.Kuningas, P.Lampinen, M.Määttä, H.Oksala, T.Oksanen, A.Putila, H.Saha, K.Salonen, H.Tauriainen, S.Tulokas |
| Tiirismaa Health Center, Hollola | T.Kivelä, L.Petlin, L.Savolainen |
| Turku Health Center | A.Artukka, I.Hämäläinen, L.Lehtinen, E.Pyysalo, H.Virtamo, M.Viinikkala, |
| M.Vähätalo | |
| Turku University Central Hospital | K.Breitholz, R.Eskola, K.Metsärinne, U.Pietilä, P.Saarinen, R.Tuominen, S.Äyräpää |
| Vaajakoski Health Center | K.Mäkinen, P.Sopanen |
| Valkeakoski Regional Hospital | S.Ojanen, E.Valtonen, H.Ylönen, M.Rautiainen, T.Immonen |
| Vammala Regional Hospital | I.Isomäki, R.Kroneld, L.Mustaniemi, M.Tapiolinna-Mäkelä |
| Vasa Central Hospital | S.Bergkulla, U.Hautamäki, V-A.Myllyniemi, I.Rusk |
